# Incidence of New Onset Diabetes Mellitus Secondary to Acute Pancreatitis: A Systematic Review and Meta-Analysis

**DOI:** 10.3389/fphys.2019.00637

**Published:** 2019-05-31

**Authors:** Mengmeng Zhi, Xiangyun Zhu, Aurelia Lugea, Richard T. Waldron, Stephen J. Pandol, Ling Li

**Affiliations:** ^1^Department of Endocrinology, Affiliated ZhongDa Hospital, School of Medicine, Southeast University, Nanjing, China; ^2^Division of Gastroenterology, Department of Medicine, Cedars-Sinai Medical Center, Los Angeles, CA, United States; ^3^Institute of Pancreas, Southeast University, Nanjing, China

**Keywords:** diabetes mellitus, acute pancreatitis, severity, necrosis, etiology

## Abstract

**Background and Aims:** Patients who have an episode of acute pancreatitis (AP) frequently develop diabetes mellitus (DM) over time. The reported incidence of DM after AP varies depending on the severity, etiology and the extent of pancreatic necrosis during AP. We performed a systematic review to determine the incidence of new-onset DM after AP episode (s), and compared the rate of DM in AP patients based upon different disease characteristics.

**Methods:** A total of 31 relevant studies with 13894 subjects were collected from Medline, Embase, and Web of Science. Stata 15 software was used for data analyses in the meta-analysis.

**Results:** The random-effects pooled incidence was 23.0% for DM (95% CI 16.0–31.0%) and 15.0% (95% CI 9.0–23.0%) for DM treated with insulin. We noted substantial heterogeneity in incidence estimates for DM and DM treated with insulin (*I*^2^ = 95.61 and 71.78%; both *p* < 0·001). The DM incidence was higher in the populations that had a severe AP (SAP) episode than in those with mild acute pancreatitis (MAP) (39 vs. 14%). Patients that displayed pancreatic necrosis during the AP attack(s) had a higher frequency of DM than those without necrosis (37 vs. 11%). In addition, the pooled incidence of DM was higher after alcoholic compared to biliary AP (28 vs. 12%). The incidence of insulin use after SAP and alcoholic AP was 21 and 18%, respectively, with very low heterogeneities. According to duration of follow-up, the pooled rate of DM and insulin use within 5 years after AP was 20 and 14%, while the rate associated with follow-up duration of more than 5 years was elevated to 37 and 25%, respectively. On meta-regression, year of publication, male proportion, age at DM test, and duration of follow-up were neither positively nor negatively associated with the incidence of DM and DM treated with insulin in patients who had a prior AP attack.

**Conclusion:** Patients with AP developed DM after discharge from hospital with a frequency of about 23%. SAP, alcoholic AP and acute necrotizing pancreatitis (ANP) were associated with increased incidence of DM. Assessments of severity, etiology, and pancreatic necrosis are critical for predicting DM development after AP.

## Introduction

The exocrine and endocrine components comprise about 90 and 2–5%, respectively, of the pancreatic mass. Disorders of the exocrine pancreas including pancreatitis and pancreatic cancer can lead to endocrine dysfunction and abnormal glucose metabolism. The American Diabetes Association and the World Health Organization classified pancreatogenic, pancreoprivic, or apancreatic diabetes mellitus (DM) as type 3c DM. (Expert Committee on the and Classification of Diabetes, 2003; American Diabetes, 2011) Type 3c diabetes is not a single entity as it results from several different exocrine pancreatic diseases including acute, relapsing, and chronic pancreatitis of any etiology, hemochromatosis, cystic fibrosis, fibrocalculous pancreatopathy, pancreatic trauma, pancreatectomy, pancreatic agenesis, and pancreatic cancer (Woodmansey et al., 2017).

Acute pancreatitis (AP) has been reported to cause DM (Lee et al., 2016; Pendharkar et al., 2017; Tu et al., 2017, 2018). However, the data on the incidence of diabetes after AP is controversial, ranging from rare cases to more than half of all patients developing DM (Angelini et al., 1984; Doepel et al., 1993; Halonen et al., 2003; Pelli et al., 2009; Umapathy et al., 2016). Few studies reported progressive improvements (Angelini et al., 1984; Ibars et al., 2002; Shen et al., 2015) or even complete recovery (Ibars et al., 2002) of abnormal glucose metabolism after one episode of AP, while most studies showed sustained impairments of pancreatic endocrine function after attacks of AP. The reasons for such huge variations between studies are attributable to inclusion of heterogenous groups of patients (severe and mild AP, AP with and without pancreatic necrosis) as well as various follow-up periods and the inclusion of patients with and without pancreatic surgery. The severity of AP appears to correlate with the magnitude of the resulting endocrine pancreatic dysfunction. A study by Garip et al. (2013) showed that endocrine dysfunction was present in 56.4% of patients after severe AP (SAP) but only in 23.2% after mild AP (MAP). However, other studies (Wu et al., 2011; Ho et al., 2015; Nikkola et al., 2017) found that DM onset did not differ significantly between the SAP and MAP groups. The criteria to define AP severity include the presence and extent of pancreatic necrosis which reflects the pancreas local situation, and aspects of system organ dysfunction. Tu et al. (2017) reported that AP patients with pancreatic necrosis had much higher incidence of DM later on compared to those who had no pancreatic necrosis. Moreover, in the group of patients with pancreatic necrosis, the rate of DM positively correlated with the area of necrosis. This study also demonstrated that the occurrence of DM continued to increase for a long time after AP, thus the risk became much greater in those patients with more than 5 years' follow-up.

Pancreatic procedures including pancreas resection and necrosectomy in SAP patients, have an obvious effect on the incidence of DM. Nordback and Auvinen (1985) observed a very high incidence of DM in 92% of SAP patients after pancreatic necrosectomy. Similarly, Sabater et al. (2004) showed that patients undergoing necrosectomy had higher incidence of pancreatic endocrine deficiency (75 vs. 26%) in long-term follow up.

Because of the reported variation in DM incidence, there is not currently a consistent model designed to predict the probability of DM after AP. To address this unmet medical need, we undertook a systematic review and meta-analysis to identify the pancreatitis characteristics that account for the variation in DM incidence after AP.

## Methods

### Search Strategy and Selection Criteria

The Preferred Reporting Items for Systematic Reviews and Meta-Analyses (PRISMA) guidelines (Liberati et al., 2009) were used to perform the review. We searched Medline, Embase and Web of Science to identify reports for our study. The search included reports from 1960 to June 30, 2018. We used search terms AP (“acute pancreatitis” or “pancreatitis, acute”) combined with “endocrine function” OR “endocrine insufficiency” OR “impaired glucose tolerance” OR “glucose homeostasis” OR “diabetes mellitus” OR “prediabetic state” OR “type 2 diabetes mellitus” OR “type 1 diabetes mellitus” OR “adult onset diabetes mellitus” OR “maturity onset diabetes” OR “non-insulin dependent diabetes” OR “insulin dependent diabetes.” The search was limited to English-language publications and human studies.

### Inclusion Criteria

1. Age equal to or greater than 18 years.
2. Measurements of glucose metabolism in AP patients were performed after more than one month from hospital discharge following episode (s) of AP.
3. Absence of a history of pre-existing pre-diabetes or diabetes before the AP episode.
4. The reports provided standard diagnosis methods for AP.
5. The reports included incidence rates or raw data to calculate the rates.

### Exclusion Criteria

1. Reports that focused specifically on either AP patients with pancreatic surgery, hereditary pancreatitis or autoimmune pancreatitis.
2. Reports in which the number of DM patients were unavailable.
3. Studies where less than 50% of the patients provided information during the follow-up or there was no report on the percentage of patients providing data during follow-up.

### Quality Assessment and Data Extraction

Titles and abstracts of the retrieved studies were scanned by two authors (MMZ reviewed all abstracts, and a second review was performed by MMZ and XYZ) to exclude irrelevant studies. MMZ and XYZ then read in detail the full text of the pre-selected articles to determine whether the studies met inclusion criteria. Reference lists of the selected articles were examined to avoid omission of any papers in the field. The corresponding authors were consulted to seek more information if required. The two authors undertook selection of studies, data extraction and quality assessment work independent of each other with all papers.

Data on the following variables were extracted: (1) year of publication (2) study design (e.g., cohort, case-control study) (3) method of sample selection (4) number and age of participants (5) proportion of male subjects (6) number of AP episodes (7) definition of AP and classification of its severity (8) criteria used to diagnose prediabetes and DM (9) etiology of AP (i.e., biliary, alcohol, other) and number of AP episodes due to each etiology (10) duration of follow-up period (11) glucose homeostasis test employed, (12) onset of prediabetes, DM and DM treated with insulin (13) number of subjects with acute necrotizing pancreatitis (ANP) and (14) occurrence of both abnormal glucose metabolism and ANP (15) presence of surgeries including pancreatic resection, necrosectomy, and lavage (16) presence of exocrine insufficiency and its measurement. If necessary, further clarifications were sought from the authors of relevant studies.

### Participants' Key Characteristics and Definitions

AP: AP was confirmed when 2 out of the 3 measures were fulfilled: (1) typical abdominal pain, (2) serum amylase and/or lipase >3 times the upper limit of normal, and/or (3) characteristic findings from abdominal imaging.

Because different definitions of diabetes were used during the years of the reports, we used the following in our review: individuals with FBG ≥7.8 mmol/L or 2 h OGTT ≥11.1 mmol/L or FBG ≥7.0 mmol/L and/or treatment with insulin, oral hypoglycemic drugs or specific dietary management.

The severity classifications of acute pancreatitis have changed over the years: (1) 1974: Ranson's Criteria (Ranson et al., 1974); (2) 1981: Acute Physiology and Chronic Health Examination (APACHE) II score ≥8 (Larvin and McMahon, 1989); (3) 1990: Balthazar computed tomography severity index (CTSI) (Balthazar et al., 1990); (4) 1992: The Atlanta Classification of acute pancreatitis (Bradley, 1993) (5) 2008: Bedside index for severity in AP (BISAP) score (Wu et al., 2008); (6) 2012: The revised Atlanta Classification of acute pancreatitis (Sarr, 2013). Most of the studies included in this review define the severity of AP with Atlanta criteria and revised Atlanta criteria, 2 with Ranson's Criteria (Malecka-Panas et al., 2002; Hochman et al., 2006) and 1 (Yasuda et al., 2008) with APACHE II score system. Further, the participants were categorized as “severe” if they had hemorrhagic AP or necrotizing AP, or met the SAP definition of Japanese severity score (Ogawa et al., 2002). The remaining subjects were considered as “mild.”

Acute necrotizing pancreatitis was determined based on contrast-enhanced CT scan, histology, surgery or medical records.

Pancreatic surgery was noted when the patient underwent pancreatic resection, necrosectomy with peritoneal lavage, retroperitoneal drainage and lavage. Surgeries not related to pancreas (e.g., cholecystectomy, cesarean section, and others) were not recorded as surgery for the purposes of this study.

Recurrent AP was recorded in patients with one or more episodes of confirmed AP since their first AP attack. Those patients with only one confirmed episode of AP were recorded as no recurrence or one single attack of AP.

Exocrine pancreatic insufficiency was determined by using the Fecal elastase (FE-1) test. Abnormal exocrine pancreatic function was defined as Elastase ≤ 200 μg/g stool. Patients with abnormal exocrine pancreatic function tested using the secretin-cerulein test according to Malfertheiner Classification were also deemed to be positive.

### Data Analysis

Pooled incidence estimates were calculated by the variance-stabilizing double arcsine transformation to generate approximation to the normal distribution, because binary data with low incidence existed in the studies included in the present review (Freeman and Tukey, 1950). With double arcsine transformation, the transformed rates were weighted very slightly toward 50%, and the incidence of zero can thus be analyzed to give the combined proportion. Meanwhile, 95% CIs were calculated by the Wilson method (Newcombe, 1998). We used random-effects models for summary statistics by STATA 15.0. The critical appraisal tool of Munn et al (Munn et al., 2014) was used to grade the quality of our incidence studies (Supplementary Table 1). The heterogeneity between studies was estimated with the *I*^2^ statistic, with values of 25, 50, and 75% showing low, moderate, and high degrees of heterogeneity, respectively (Higgins et al., 2003). The research bias in the publications was evaluated with the Egger's (Sterne et al., 2001) test. To evaluate if the results were stable and reliable, a sensitivity analysis was performed after excluding two studies [one with a large population (Ho et al., 2015), and another one with a small population (Seligson et al., 1982)] from the prediabetic and/or diabetic group(s). Forest plots were generated to show incidence proportions. Studies were grouped according to the etiology and severity of AP, and whether the participants had necrosis, recurrence, surgery, or pancreatic exocrine insufficiency. We defined subgroups of etiology (alcoholic, biliary vs. others), severity (MAP vs. SAP), and necrosis (ANP vs. non-ANP). Potential sources of heterogeneity were further investigated by arranging groups of studies according to potentially relevant features and by meta-regression analysis, which attempts to relate differences in effect sizes to study features (Thompson and Higgins, 2002). Four univariate meta-regression analyses (*post hoc*) were carried out to examine the relation of incidence of diabetes in AP to 4 factors: (1) publication year, (2) age, (3) male proportion, and (4) duration of following up. All these factors could explain the variance of diabetes incidence. All statistical analyses were performed using STATA (version 15.0) with the commands metaprop (for random-effects meta-analysis specifying two variables: double-arcsine-transformed incidence and Wilson CIs) and metareg (for metaregression).

## Results

**Figure 1:**
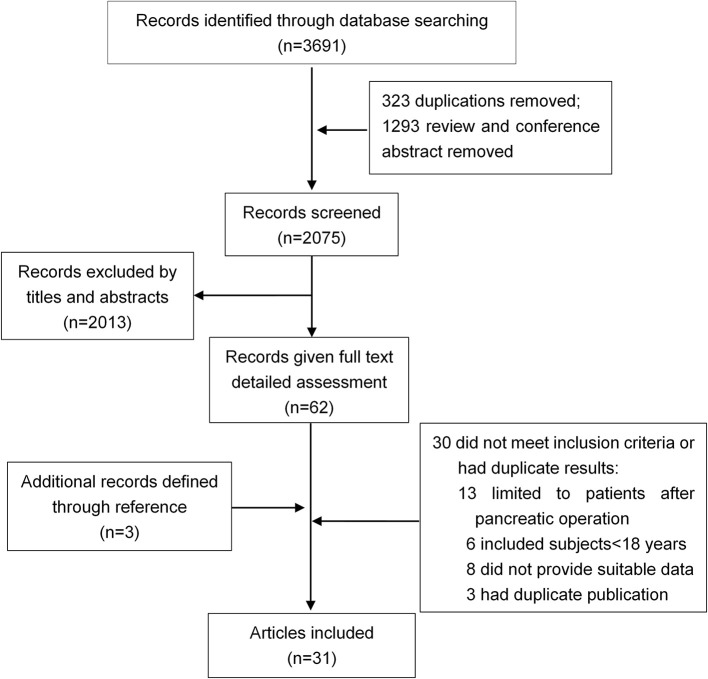
Flowchart for study inclusion.

Thirty-one studies were selected for inclusion (Figure 1). We scanned a total of 3691 publications. After removal of duplicates and initial screening of titles and abstracts, 62 papers were reviewed by reading the full text. After exclusion of ineligible reports, the final sample comprised 31 studies (*n* = 13,894 subjects) published between June 1, 1946, and June 30, 2018, with a total of 31 (13894) on DM (Ohlsen, 1968; Johansen and Ornsholt, 1972; Olszewski et al., 1978; Seligson et al., 1982; Angelini et al., 1984, 1993; Eriksson et al., 1992; Doepel et al., 1993; Malecka-Panas et al., 1996, 2002; Appelros et al., 2001; Ibars et al., 2002; Boreham and Ammori, 2003; Halonen et al., 2003; Szentkereszty et al., 2004; Hochman et al., 2006; Kaya et al., 2007; Yasuda et al., 2008; Gupta et al., 2009; Pelli et al., 2009; Andersson et al., 2010; Uomo et al., 2010; Garip et al., 2013; Vujasinovic et al., 2014; Chandrasekaran et al., 2015; Ho et al., 2015; Winter Gasparoto et al., 2015; Umapathy et al., 2016; Vipperla et al., 2016; Nikkola et al., 2017; Tu et al., 2017), and 10 studies (428) on DM treated with insulin (Ohlsen, 1968; Eriksson et al., 1992; Doepel et al., 1993; Appelros et al., 2001; Malecka-Panas et al., 2002; Boreham and Ammori, 2003; Hochman et al., 2006; Yasuda et al., 2008; Gupta et al., 2009; Chandrasekaran et al., 2015) (Table 1). Sample sizes ranged from 9 to 12284, with most studies including sample sizes between 15 and 150. One study had a minimum size of 9 (Seligson et al., 1982), and other one a maximum size of 12284 (Ho et al., 2015). The critical appraisal tool of Munn et al(Munn et al., 2014) was used to grade the quality of our incidence studies, the results are shown in Supplementary Table 1. We examined the incidence of DM in studies fulfilling all quality criteria. In these 14 studies, the DM incidence was 23% (95% CI 13–35%) (Supplementary Figure 1).

**Table 1:** Demographic and clinical characteristics of included studies.

**References**	Duration of follow-up^*^	**Age**	**Male proportion (%)**	**NO. of AP episodes per person**	**Criteria for SAP**	**Glucose measurement**	Total no. of subjects^**^	**No. of DM**	**No. of insulin use**
Ohlsen, 1968	NR	51	39.1	1	NR	IV GTT	23	0	0
Johansen and Ornsholt, 1972	24	37	41.7	>1	NR	OGTT	22	4	NR
Olszewski et al., 1978	12	41	72	NR	NR	OGTT, BI	25	7	NR
Seligson et al., 1982	63	55	77.8	>1	NR	OGTT	9	2	NR
Angelini et al., 1984	25, 40	NR	88.9	1	NR	OGTT	19	1	NR
Eriksson et al., 1992	74	43	66.7	>1	NR	OGTT	36	19	9
Angelini et al., 1993	53	NR	NR	>1	NR	OGTT	118	9	NR
Doepel et al., 1993	74	49	67.6	NR	Multiple organ failure with hemorrhagic and/or necrotic pancreatitis	BG, HbA1c, OGTT	37	20	9
Malecka-Panas et al., 1996	48 to 84	43.5	70.2	1	NR	OGTT	47	8	NR
Appelros et al., 2001	83	59	65.8	>1	Atlanta criteria	Questionnaire, BG, HbA1c	35	15	9
Ibars et al., 2002	1, 6, 12	62	27	NR	Atlanta criteria	OGTT, Arginine test	55	6	NR
Malecka-Panas et al., 2002	56	47	67.1	>1	Ranson criteria	OGTT, Insulin test	82	13	6
Boreham and Ammori, 2003	3	55, median	56.5	1	Atlanta criteria	FBG	23	4	1
Halonen et al., 2003	66	44	82.8	>1	Atlanta criteria	Questionnaire	145	68	NR
Szentkereszty et al., 2004	38	46	76	>1	NR	Questionnaire	22	3	NR
Hochman et al., 2006	24, 36	62	57.1	NR	Ranson criteria	Questionnaire	25	8	5
Kaya et al., 2007	12	55	51.3	NR	NR	OGTT	112	13	NR
Yasuda et al., 2008	56	52	81.3	>1	JSS	FBG	41	16	4
Pelli et al., 2009	23, median	49, median	87	>1	Atlanta criteria	OGTT, HbA1c	46	5	NR
Gupta et al., 2009	31	38	80	>1	Atlanta criteria	FBG, PBG, OGTT	30	6	6
Andersson et al., 2010	45	59	40	1	Atlanta criteria	FBG, Insulin, OGTT	39	9	NR
Uomo et al., 2010	179	48	42.5	NR	NR	FBG, OGTT	38	6	NR
Garip et al., 2013	32	56.5	53.2	NR	APACHE II ≥ 8	FBG, OGTT	96	33	NR
Vujasinovic et al., 2014	32	56.5	65	>1	NR	FBG, OGTT	100	14	NR
Chandrasekaran et al., 2015	26.2	36.8	80.6	>1	Atlanta criteria	OGTT	35	17	12
Ho et al., 2015	>24	50.2	70.6	>1	Atlanta criteria	Medical reports	12284	618	NR
Winter Gasparoto et al., 2015	34.8	56.2	48	1	NR	OGTT, HOMA-IR	16	5	NR
Umapathy et al., 2016	>12	50.7	68	>1	NR	Medical reports	73	33	NR
Vipperla et al., 2016	34.5	53.4	63	>1	NR	Medical reports	101	28	NR
Nikkola et al., 2017	126	48	90	>1	Atlanta criteria	Medical reports	47	7	NR
Tu et al., 2017	42.9	47.2	66.4	>1	Atlanta criteria	OGTT, HbA1c	113	34	NR

*AP, Acute pancreatitis; SAP, Severe acute pancreatitis; GTT, Glucose tolerance test; OGTT, Oral glucose tolerance test; BG, Blood glucose; FBG, Fasting blood glucose; HbA1c, Glycohaemoglobin A1c; NR, Not reported; JSS, Japanese severity score*.

* *The duration of following up was presented as average if not stated as median or a range*.

** *Total number of subjects excluding pre-existing diabetes before acute pancreatitis*.

No evidence of funnel plot asymmetry for DM (excluding the study with 12284 subjects) and DM treated with insulin was found (*p*-values of 0.328 and 0.169 for Egger's test, respectively; Supplementary Figure 2), indicating a lack of publication bias. When we included the large study with 12284 subjects, there was an obvious publication bias by Egger's test.

**Figure 2:**
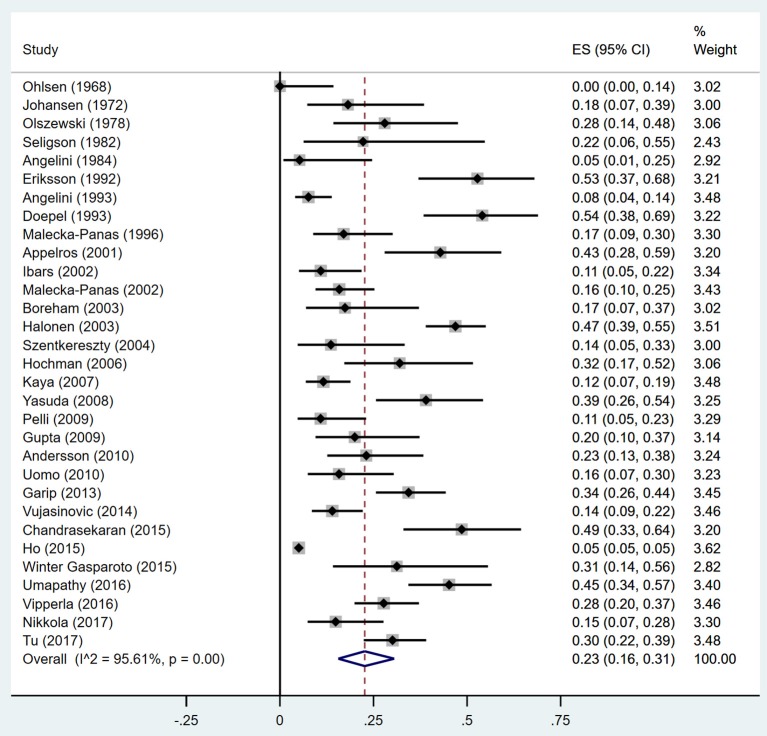
Incidence of diabetes after acute pancreatitis. CI, confidence interval.

**Figure 3:**
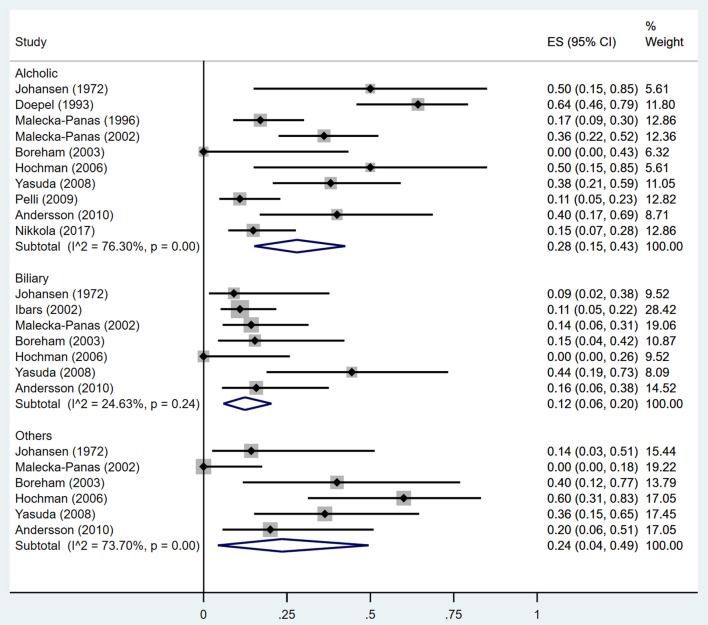
Incidence of diabetes after acute pancreatitis caused by different etiologies. CI, confidence interval.

**Figure 4:**
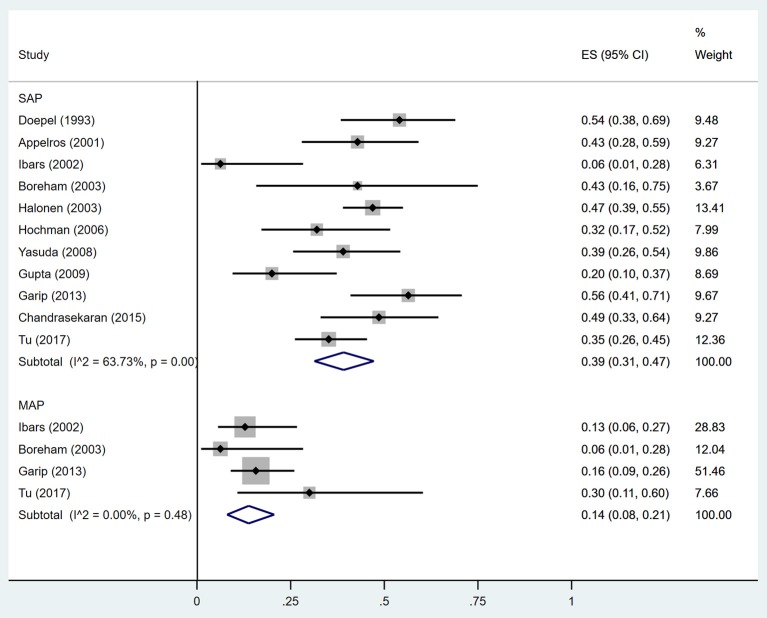
Incidence of diabetes after acute pancreatitis of different severities. SAP, Severe acute pancreatitis; MAP, Mild acute pancreatitis; CI, confidence interval.

**Figure 5:**
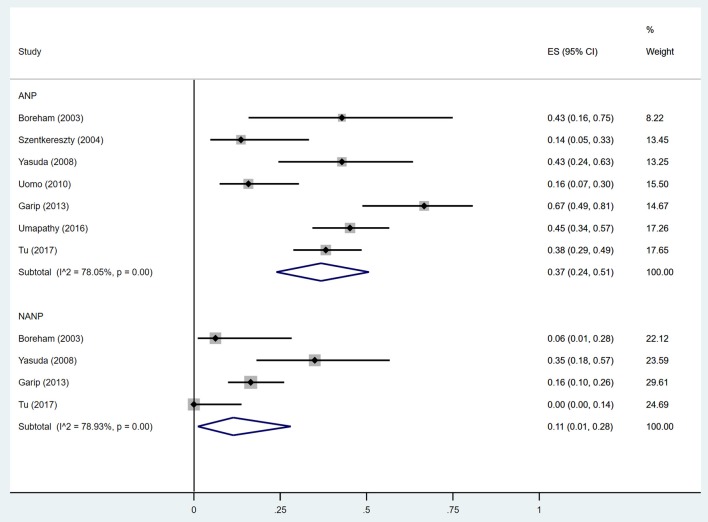
Incidence of diabetes after acute pancreatitis with and without necrosis. ANP, acute necrotizing pancreatitis; NANP, Non-ANP; CI, confidence interval.

**Figure 6:**
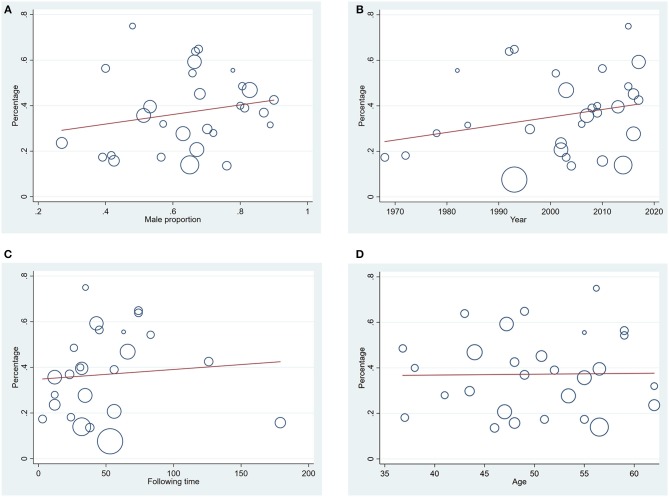
**(A)** Meta-regression of relation between male proportion and diabetes incidence after acute pancreatitis (*P* = 0.284); **(B)** Meta-regression of relation between year of publication of the studies and diabetes incidence after acute pancreatitis (*P* = 0.173); **(C)** Meta-regression of relation between duration of following up and diabetes incidence after acute pancreatitis (*P* = 0.671); **(D)** Meta-regression of relation between mean age in the general population and diabetes incidence after acute pancreatitis (*P* = 0.938).

Estimates of the incidence of DM after AP ranged from 0 to 54.0% (Figure 2); heterogeneity was pronounced (*I*^2^ = 95.61%, *P* < 0.001). The random-effects pooled incidence was 23.0% (95% CI 16.0–31.0%). Removal of either or both of the two studies with the lowest and highest populations did not affect the overall pooled estimate or the level of heterogeneity. The result of sensitivity analysis was shown in Supplementary Table 2. Data were available for DM incidence among subjects with alcoholic etiology in 10 studies (Johansen and Ornsholt, 1972; Doepel et al., 1993; Malecka-Panas et al., 1996, 2002; Boreham and Ammori, 2003; Hochman et al., 2006; Yasuda et al., 2008; Pelli et al., 2009; Andersson et al., 2010; Nikkola et al., 2017), biliary etiology in 7 studies (Johansen and Ornsholt, 1972; Ibars et al., 2002; Malecka-Panas et al., 2002; Boreham and Ammori, 2003; Hochman et al., 2006; Yasuda et al., 2008; Andersson et al., 2010) and other etiology in 6 studies (Johansen and Ornsholt, 1972; Malecka-Panas et al., 2002; Boreham and Ammori, 2003; Hochman et al., 2006; Yasuda et al., 2008; Andersson et al., 2010) (Table 2 and Supplementary Table 3). Incidence of DM in subjects with and without previous history of ANP was reported in 7 (Boreham and Ammori, 2003; Szentkereszty et al., 2004; Yasuda et al., 2008; Uomo et al., 2010; Garip et al., 2013; Umapathy et al., 2016; Tu et al., 2017) and 4 studies (Boreham and Ammori, 2003; Yasuda et al., 2008; Garip et al., 2013; Tu et al., 2017). Besides, 11 (Doepel et al., 1993; Appelros et al., 2001; Ibars et al., 2002; Boreham and Ammori, 2003; Halonen et al., 2003; Hochman et al., 2006; Yasuda et al., 2008; Gupta et al., 2009; Garip et al., 2013; Chandrasekaran et al., 2015; Tu et al., 2017) and 4 studies (Ibars et al., 2002; Boreham and Ammori, 2003; Garip et al., 2013; Tu et al., 2017) reported data of DM morbidity after SAP and MAP (Table 2). In the subgroup analyses, pooled incidence of DM after alcoholic, biliary or AP due to other etiology was, respectively 28% (*I*^2^ = 76.3%, *P* < 0.001), 12% (*I*^2^ = 24.6%, *P* = 0.24) and 24% (*I*^2^ = 73.7%, *P* < 0.001) (Figure 3). In addition, combined incidence of DM after SAP and MAP was 39% (*I*^2^ = 63.73%, *P* < 0.001) and 14% (*I*^2^ = 0 %, *P* = 0.48), respectively (Figure 4). Subjects who developed DM after AP with and without necrosis showed different integrated rates of 37% (*I*^2^ = 78.1%, *P* < 0.001) and 11% (*I*^2^ = 78.9%, *P* < 0.001), respectively (Figure 5). Concerning duration of follow-up (< 5 years or > 5 years), pooled incidence of DM within 5 years was 20%, while that over 5 years was increased to 37% (Table 3). In the meta-regression, we found no association between the incidence of DM after AP and the proportion of the male patients (*P* = 0.284; Figure 6A), year of publication (*P* = 0.173; Figure 6B), duration of following up (*P* = 0.671; Figure 6C), or mean age (*P* = 0.938; Figure 6D).

**Table 2:** Diabetes occurrence of AP subjects with different characteristics.

**Study**	**MAP**		**SAP**		**ANP**		**NANP**		**Alcoholic**		**Biliary**	
	**DM**	**Total no**.	**DM**	**Total no**.	**DM**	**Total no**.	**DM**	**Total no**.	**DM**	**Total no**.	**DM**	**Total no**.
Johansen	—	—	—	—	—	—	—	—	2	4	1	11
Doepel	—	—	20	37	—	—	—	—	18	28	—	—
Malecka-Panas	—	—	—	—	—	—	—	—	8	47	—	—
Appelros	—	—	15	35	—	—	—	—	—	—	—	—
Ibars	5	39	1	16	—	—	—	—	—	—	6	55
Malecka-Panas	—	—	—	—	—	—	—	—	13	36	4	28
Boreham	1	16	3	7	3	7	1	16	0	5	2	13
Halonen	—	—	68	145	—	—	—	—	—	—	—	—
Szentkereszty	—	—	—	—	3	22	—	—	—	—	—	—
Hochman	—	—	8	25	—	—	—	—	2	4	0	11
Kaya	—	—	—	—	—	—	—	—	—	—	—	—
Yasuda	—	—	16	41	9	21	7	20	8	21	4	9
Pelli	—	—	—	—	—	—	—	—	5	46	—	—
Gupta	—	—	6	30	—	—	—	—	—	—	—	—
Andersson	—	—	—	—	—	—	—	—	4	10	3	19
Uomo	—	—	—	—	6	38	—	—	—	—	—	—
Garip	11	70	22	39	20	30	13	79	—	—	—	—
Chandrasekaran	—	—	17	35	—	—	—	—	—	—	—	—
Umapathy	—	—	—	—	33	73	—	—	—	—	—	—
Nikkola	—	—	—	—	—	—	—	—	7	47	—	—
Tu	3	10	32	91	34	89	0	24	—	—	—	—

*AP, Acute pancreatitis; MAP, Mild acute pancreatitis; SAP, Severe acute pancreatitis; ANP, Acute necrotizing pancreatitis; NANP, Non-ANP; DM, Diabetes mellitus*.

**Table 3:** Incidence of diabetes and insulin usage after acute pancreatitis according to different duration of following up.

**Duration of following up**	**DM**			**Insulin usage**		
	**Studies**	**Incidence (95% CI)**	**Heterogeneity**	**Studies**	**Incidence (95% CI)**	**Heterogeneity**
< 5 years	23	0.20 (0.15, 0.26)	80.37%	6	0.14 (0.06, 0.21)	0.00%
≥ 5 years	6	0.37 (0.23, 0.52)	85.19%	3	0.25 (0.17, 0.33)	67.10%

*DM, Diabetes mellitus; CI, confidence interval*.

**Figure 7:**
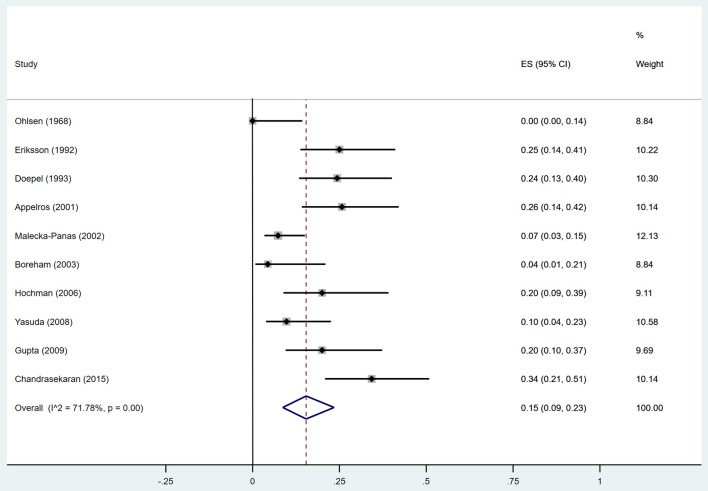
Incidence of insulin-treated diabetes after acute pancreatitis. CI, confidence interval.

Estimates of the incidence of insulin-treated DM after AP ranged from 0 to 34.0% (Figure 7); The random-effects pooled incidence was 15.0% (95% CI 9.0–23.0%). There was moderate statistical heterogeneity between studies (*I*^2^ = 71.78%, *P* < 0.001). Therefore, we performed sensitivity analyses, subgroup analyses, and meta-regression to examine this heterogeneity. We constrained our analysis to studies evaluating insulin usage after SAP (Doepel et al., 1993; Appelros et al., 2001; Boreham and Ammori, 2003; Hochman et al., 2006; Yasuda et al., 2008; Gupta et al., 2009; Chandrasekaran et al., 2015) or alcoholic AP (Doepel et al., 1993; Boreham and Ammori, 2003; Hochman et al., 2006), finding pooled incidence of 21% (95% CI 15 to 28%) and 18% (95% CI 5 to 35%), respectively, with low heterogeneity between these studies (*I*^2^ = 19.99%, *P* = 0.28; *I*^2^ = 3.01%, *P* = 0.36) (Supplementary Figures 3, 4). According to duration of follow-up (< 5 years and > 5 years), pooled incidence of the treatment with insulin within 5 years was 14%, while that over 5 years was elevated to 25% (Table 3). The meta-regression could not demonstrate any associations between the insulin-treated DM and factors including proportion of the male, year of publication, duration of following up and mean age (Supplementary Table 4).

## Discussion

Findings from this systematic review and meta-analysis suggest that DM is an important problem for AP patients, although there is wide variation in the incidence of DM between populations from different subgroups. A previous study (Das et al., 2014) reported the pooled estimates of the incidence of endocrine dysfunction (both prediabetes and diabetes) in 40% after a first attack of AP. In this review, we increased the number of included studies and enlarged the population of AP, which could further strengthen the reliability of the result of DM rate after AP. Additionally, we restrictedly focused on the occurrence of diabetes only, finding a similar incidence of DM after AP to the result of the prior meta-analysis in about 23%. What's particularly different from the prior meta-analysis is that we compared the DM rate among AP subjects with various severity and etiology, with and without the presence of pancreatic necrosis. Those subjects with SAP, alcoholic AP and ANP have a DM incidence after the AP attack of 39, 28, and 37% respectively compared with lower DM rates of 14, 12, and 11% in MAP, biliary AP and non-ANP, respectively. This finding indicates the severity, etiology, and necrosis are crucial factors in predicting new-onset DM after AP.

DM secondary to pancreatic diseases is classified as pancreatogenic diabetes (American Diabetes, 2011). Acute pancreatitis, as the most common pancreatic disorder, is more often associated with the development of pancreatic endocrine dysfunction. However, there is little information relating pancreatic exocrine function to the development of diabetes after an episode of AP. Few studies reported full pancreatic functional recovery (both exocrine and endocrine) after AP (Mitchell et al., 1983; Angelini et al., 1993), while other studies found both endocrine and exocrine insufficiency after AP attack(s) (Büchler et al., 1985; Seidensticker et al., 1995). To our knowledge, acute stress, pancreatic microcirculation disorder and excessive secretion of catecholamines after AP could result in disruption of glucose metabolism leading to a transient rise in blood glucose. During the recovery phase of AP, blood glucose levels would rapidly return to normal in most patients (Symersky et al., 2006; Kaya et al., 2007; Garip et al., 2013). However, a subset of the patients will develop DM and need prolonged antidiabetic treatments including insulin (Mentula et al., 2008; Czakó et al., 2009). One possible mechanism of DM secondary to AP could be nutrient maldigestion induced by exocrine insufficiency that causes abnormal incretin secretion and impaired insulin release from β-cells (Ebert and Creutzfeldt, 1980). Increased insulin resistance could be another explanation for abnormal carbohydrate metabolism after AP (Yeo et al., 1989; Buscher et al., 1999). These two possible mechanisms appear to be associated with classical type 2 diabetes (Balzano et al., 2014), which illustrates that T3cDM might be a heterogeneous disorder strongly overlapping with type 2 diabetes. In addition, the loss of pancreatic β cells caused by necrosis is considered to be a main cause of DM after AP, especially in those subjects with necrosectomy. A prior study (Chandrasekaran et al., 2015) made comparison between non-operative and necrosectomy group, higher incidence of abnormal glucose tolerance was also observed in patients undergoing necrosectomy. Besides, it was observed that insulin requirement was significantly higher in necrosectomy group.

ANP is reported to be associated with a higher risk of subsequent endocrine insufficiency and DM (Winter Gasparoto et al., 2015; Umapathy et al., 2016; Tu et al., 2018). One study showed that the incidence of new-onset diabetes after ANP was as high as 45% (Umapathy et al., 2016). A study by Tu et al. (2017) showed that the development of DM correlated strongly with the extent of pancreatic necrosis. The ANP patients with necrosis over 50% of the pancreas had a much higher incidence of DM than those with necrosis area < 30% (57.7 vs. 19.4%), as well as worse control of glucose as measured by HbA1C% (*P* = 0.001). They also presented that necrosis in the tail of the pancreas had more risks for DM than that in the pancreatic head or body, as more islets are distributed in the tail of the pancreas. As a result, ANP is a risk factor for DM promotion, which could account for the higher incidence of DM in 37% compared with 11% in AP patients without necrosis. A likely cause of the greater incidence of DM after ANP is a decrease in numbers of functional islet β-cells and levels of secreted insulin due to tissue destruction (Gupta et al., 2009). Furthermore, a subset of ANP patients are subjected to open necrosectomy and partial pancreatectomy as gold-standard approaches for treating ANP-related complications (Freeman et al., 2012), which may cause pancreatogenic diabetes as reported before (Tsiotos et al., 1998; Boreham and Ammori, 2003; Bavare et al., 2004; Kahl and Malfertheiner, 2004; Connor et al., 2005; Busse and Ainsworth, 2015; Kapoor, 2016;Roeyen et al., 2016).

Along with ANP, SAP might be another accelerating factor for developing DM after AP. Compared to mild AP, SAP is linked to higher degree of pancreatic injury, which may promote endocrine insufficiency. However, data on the expediting impact of SAP on DM development is equivocal. Several studies reported no association between the severity of AP and the rate of subsequent DM (Ho et al., 2015; Nikkola et al., 2017), while others concluded that SAP led to increased occurrence of impaired glucose metabolism (Uomo et al., 2010; Garip et al., 2013; Vipperla et al., 2016). The severity of AP depends on both pancreatic necrosis (reflecting the pancreas local circumstance) and organ dysfunction, and surgical intervention is a common choice for pancreatic necrosis. Long-term outcomes in patients with SAP managed by surgical and non-surgical treatments were compared by Chandrasekaran et al. (2015). The authors found a higher incidence of endocrine dysfunction in AP patients undergoing necrosectomy. These considerations suggest it may be difficult to dissociate the effects of pancreatic necrosis vs. surgical intervention.

A greater rate of DM after alcoholic compared to biliary AP was discovered in our study, which is consistent with a nationwide population-based study by Ho et al. (2015). Alcohol abuse can damage the pancreas directly or via its metabolites. Studies demonstrate that alcohol causes pancreatitis via precipitation of secreted proteins (protein plug formation) within small pancreatic ductules leading to pancreas atrophy and fibrosis, and ultimately promotes premature intracellular digestive enzyme activation leading to autodigestive injury (Sarles, 1971, 1974; Guy et al., 1983; Apte et al., 1995). Moreover, alcohol and its metabolites, cytokines and growth factors released during alcohol-induced pancreatic necroinflammation can activate pancreatic stellate cells, which will be responsible for the ongoing inflammation and fibrosis of the pancreas (Apte et al., 2010). The sustained exocrine damage caused by pancreatic autodigestive injury and pancreatic fibrosis are both risk factors for DM development. Meanwhile, pancreatic endocrine dysfunction is considered to be associated with recurrent AP (Sand and Nordback, 2009; Ho et al., 2015; Nikkola et al., 2017). Nikkola et al. (2017) discovered that half of the patients with recurrent AP developed new pancreatogenic diabetes in a median of 4.3 years after the initial attack, and only the patients with recurrent AP episodes developed pancreatogenic diabetes. Alcoholic pancreatitis is the most likely form of AP to be recurrent, which suggests that patients with alcoholic AP are at a higher risk of developing diabetes associated with recurrent episodes of pancreatitis.

In terms of the trend of DM occurrence with time, there was a significant increase in the incidence of diabetes discovered in the present review, which is similar to findings in a previous study (Das et al., 2014). An observational study by Tu et al. (2017) showed that the value of HbA1C gradually increased over time after AP indicating that endocrine pancreatic function decreases over time after AP. However, due to the fact that T3cDM is a heterogeneous entity strongly overlapping with type 2 diabetes, it is difficult to establish conclusively whether it is caused by AP or is following its own natural course. On the other hand, individuals may suffer from further attacks of AP during 5 years, which apparently contribute to an increased incidence of DM with duration of follow-up. Several studies included in our review indeed reported that some patients experienced recurrent AP (Angelini et al., 1984; Eriksson et al., 1992; Appelros et al., 2001; Malecka-Panas et al., 2002; Halonen et al., 2003; Szentkereszty et al., 2004; Yasuda et al., 2008; Gupta et al., 2009; Pelli et al., 2009; Ho et al., 2015; Nikkola et al., 2017; Tu et al., 2017), which might increase the rate of DM with time.

Our analysis observed significant heterogeneity that could not be fully explained by the examined characteristics. Potential sources of heterogeneity were further investigated by arranging subgroups according to etiology (alcoholic, biliary vs. others), severity (MAP vs. SAP), and necrosis (ANP vs. non-ANP) and by meta-regression analysis. The subgroup analysis decreased the heterogeneity to a degree, especially in the biliary AP and MAP group. Meta-regressions were performed to examine the interaction between age, sex, year of publication, duration of follow-up and incidence of DM after AP, and none of these explained the heterogeneity. Considering the possibility that publication bias could be a cause for the high heterogeneity, we made funnel plots finding no evidence of funnel plot asymmetry for DM after excluding the study with 12284 subjects. When we included the study with 12284 samples, there was an obvious publication bias by Egger's test. However, when we removed the study with huge number of patients, neither of the heterogeneity or the incidence changed significantly.

Strengths of the present study include a large pool of identified studies drawn from 3 different databases (Medline, EMBASE and Web of science). This large number of studies enabled us to relate the diabetes outcome to subsets of patients with different severities and etiologies. The large pool of studies also afforded meta-regression with examination of important demographic variables as interacting factors. We were able to use high quality studies, excluding those of patients with pre-existing DM and others focused on pancreatic surgery. Thus, we were able to focus our work on patients with newly diagnosed DM after AP without the confounding factors related to the effect of surgical islet removal on DM development.

An observed heterogeneity that lacks sufficient explanatory factors is a significant limitation of our study that affects interpretation of the pooled estimate. Although some of our subgroup analyses involving patients in biliary AP and MAP groups would decrease heterogeneity to a low level, some other groups with high heterogeneity could not be explained by sensitivity analysis and meta-regression analysis. Such a high heterogeneity might be associated with several limitations of the study, such as differences in the number of AP attacks between the studies. Most of the studies did not indicate whether patients had multiple AP attacks. Only two studies reported that RAP was associated with the development of pancreatogenic diabetes, while another one found no correlation between the number of AP attacks and glucose tolerance abnormalities. Consequently, the effect of RAP on risk of developing DM remains unknown. Despite our exclusion of studies which only paid attention to pancreatic surgery, 15 of the studies we retained did report patients undergoing pancreatic surgical interventions including necrosectomy, peritoneal lavage, percutaneous drainage and pancreatectomy. The 4 studies which compared DM development between patients with and without pancreatic surgery found a much higher rate of DM after surgery. Hence, pancreatic surgery is a risk factor for DM and is another source of heterogeneity. Furthermore, the severity classifications of acute pancreatitis have changed over the years. Different criteria were used for the severity classification of AP in the included studies, which is particularly important to the high level of heterogeneity.

There are also some other limitations in the present study. Eleven studies included in the review focused on SAP, which broke the assumption that a conglomerate of all the studies is representation of all patients with acute pancreatitis. The fact that mild acute pancreatitis is under-represented inflated the figures for the incidence of diabetes mellitus. Even though subjects with chronic pancreatitis were excluded in the studies we chose, patients with RAP were not totally eliminated. RAP is recognized as an intermediary stage in the pathogenesis of chronic pancreatitis, and a subset of RAP patients undergo their natural course transition to chronic pancreatitis (Párniczky et al., 2016; Machicado and Yadav, 2017). Thus, potential mixture of chronic pancreatitis and RAP could influence the outcome. As reported, body mass index (BMI) highly influence the outcome of AP (Dobszai et al., 2019). The lack of data on the changes in body weight or BMI is a potential negative factor to find a causal relation between AP attacks and the rising risk of DM. Besides, most of the studies excluded patients who died from the attack of AP during the follow up, which represents a selection bias influencing the active incidence. Additionally, although the studies included in our meta-analysis claimed that they have ruled out subjects with pre-existing diabetes, measurement of HbA1C within the first 3 months after AP which could definitely exclude pre-existing DM was performed in only 4 studies. This actually impact the real incidence of DM and even the AP process.

## Conclusion

The results of our analysis show that ~1 in 5 patients with an AP episode develops DM afterwards, and the rate increases over time. In addition, the occurrence of DM after alcoholic AP, SAP, and ANP was 2 to 3 times higher than that secondary to biliary AP, MAP, and AP without necrosis. Our findings strongly highlight the importance of regular long-term follow-up for endocrine function in patients after AP, especially in those with severe, alcoholic, and necrotizing pancreatitis. The early diagnosis and treatment of endocrine impairment can also help the population prevent deterioration of pancreatic exocrine function.

## Author Contributions

MZ, SP, and LL designed the study and wrote the paper. MZ and XZ performed the work and analyzed the data. AL and RW made technical and scientific contributions, and reviewed the manuscript. All the authors approved the final version.

### Conflict of Interest Statement

The authors declare that the research was conducted in the absence of any commercial or financial relationships that could be construed as a potential conflict of interest.
