# Supplementary material for: Incidence of New Onset Diabetes Mellitus Secondary to Acute Pancreatitis: A Systematic Review and Meta-Analysis
Source: Front Physiol. 2019 May 31;10:637. doi: 10.3389/fphys.2019.00637 (PMC6558372; doi:10.3389/fphys.2019.00637)
Supplement: Supplementary file 1 [file Table_1.DOCX]

**Supplementary Materials**

**Supplementary Table 1.** Quality assessment of studies in which incidence of diabetes after acute pancreatitis reported

| Author | Study design | N |  | | | | | | | | | | | | | | |
| --- | --- | --- | --- | --- | --- | --- | --- | --- | --- | --- | --- | --- | --- | --- | --- | --- | --- |
|  |  |  | Representative sample | Appropriate recruition | | | Sample size | | Subjects described | Refuser described | AP measure | Diabetic measure | | Appropriate statistical analysis | Identified confounders | Identified subpopulation |  |
| Ohlsen | Cohort | 23 | Y | | Y | Y | | Y | | U | Y | Y | Y | | Y | N/A |  |
| Johansen | Cohort | 24 | Y | | Y | Y | | Y | | Y | Y | Y | Y | | Y | N/A |  |
| Olszewski | Cohort | 25 | Y | | Y | Y | | Y | | Y | Y | Y | Y | | Y | N/A |  |
| Seligson | Cohort | 13 | Y | | Y | U | | Y | | Y | Y | Y | Y | | Y | N/A |  |
| Angelini | Cohort | 27 | U | | Y | Y | | Y | | N/A | Y | Y | Y | | Y | N/A |  |
| Eriksson | Cohort | 36 | Y | | Y | Y | | Y | | Y | Y | Y | Y | | Y | N/A |  |
| Angelini | Cohort | 118 | Y | | Y | Y | | Y | | Y | Y | Y | Y | | Y | Y |  |
| Doepel | Cohort | 37 | Y | | Y | Y | | Y | | Y | Y | Y | Y | | Y | N/A |  |
| Malecka-Panas | Cohort | 47 | Y | | Y | Y | | Y | | U | Y | Y | Y | | Y | N/A |  |
| Appelros | Cohort | 35 | Y | | Y | Y | | Y | | Y | Y | Y | Y | | Y | N/A |  |
| Ibars | Cohort | 63 | Y | | Y | Y | | Y | | Y | Y | Y | Y | | Y | Y |  |
| Malecka-Panas | Cohort | 82 | U | | U | Y | | Y | | N/A | Y | Y | Y | | Y | Y |  |
| Boreham | Cohort | 23 | Y | | Y | Y | | Y | | Y | Y | Y | Y | | Y | Y |  |
| Halonen | Cohort | 145 | Y | | Y | Y | | Y | | Y | Y | Y | Y | | Y | Y |  |
| Szentkereszty | Cohort | 22 | Y | | U | Y | | Y | | Y | Y | Y | Y | | Y | Y |  |
| Hochman | Cohort | 25 | Y | | Y | Y | | Y | | Y | Y | Y | Y | | Y | Y |  |
| Kaya | Cohort | 199 | Y | | Y | Y | | Y | | Y | Y | Y | Y | | Y | Y |  |
| Yasuda | Cohort | 45 | Y | | Y | Y | | Y | | N/A | Y | Y | Y | | Y | Y |  |
| Pelli | Cohort | 54 | Y | | Y | Y | | Y | | N | Y | Y | Y | | Y | Y |  |
| Gupta | Cohort | 30 | Y | | Y | Y | | Y | | Y | Y | Y | Y | | Y | Y |  |
| Andersson | Cohort | 40 | Y | | Y | Y | | Y | | Y | Y | Y | Y | | Y | Y |  |
| Uomo | Cohort | 40 | Y | | Y | Y | | Y | | Y | Y | Y | Y | | Y | N/A |  |
| Garip | Cohort | 109 | Y | | Y | Y | | Y | | Y | Y | Y | Y | | Y | Y |  |
| Vujasinovic | Cohort | 100 | Y | | Y | Y | | Y | | N/A | Y | Y | Y | | Y | Y |  |
| Chandrasekaran | Cohort | 35 | Y | | Y | Y | | Y | | Y | Y | Y | Y | | Y | Y |  |
| Ho | Cohort | 12284 | Y | | Y | Y | | Y | | Y | Y | Y | Y | | Y | Y |  |
| Winter Gasparoto | Cohort | 16 | Y | | Y | Y | | Y | | N/A | Y | Y | Y | | Y | Y |  |
| Umapathy | Cohort | 167 | Y | | Y | Y | | Y | | Y | Y | Y | Y | | Y | Y |  |
| Vipperla | Cohort | 127 | Y | | U | Y | | Y | | Y | Y | Y | Y | | Y | N/A |  |
| Nikkola | Cohort | 77 | Y | | Y | Y | | Y | | Y | Y | Y | Y | | Y | Y |  |
| Tu | Cohort | 113 | Y | | Y | Y | | Y | | Y | Y | Y | Y | | Y | Y |  |

Y=Yes, N=No, U=Unclear, N/A=Not/Applicable
