# Supplementary material for: Incidence of New Onset Diabetes Mellitus Secondary to Acute Pancreatitis: A Systematic Review and Meta-Analysis
Source: Front Physiol. 2019 May 31;10:637. doi: 10.3389/fphys.2019.00637 (PMC6558372; doi:10.3389/fphys.2019.00637)
Supplement: Supplementary file 2 [file Table_2.DOCX]

**Supplementary Table 2. The result of sensitivity analysis of DM incidence.**

| Study omitted | Estimate | [95% Conf. Interval] | |
| --- | --- | --- | --- |
| Combined | 0.24390899 | 0.18894841 | 0.29886958 |
| Ohlsen | 0.243909 | 0.18894841 | 0.29886958 |
| Johansen | 0.24590854 | 0.18986408 | 0.30195302 |
| Olszewski | 0.24283436 | 0.18710186 | 0.29856688 |
| Seligson | 0.24438627 | 0.18877164 | 0.30000091 |
| Angelini | 0.25124875 | 0.19440444 | 0.30809304 |
| Eriksson | 0.23475023 | 0.18043643 | 0.28906402 |
| Angelini | 0.25173536 | 0.19168547 | 0.31178522 |
| Doepel | 0.23423502 | 0.180071 | 0.28839904 |
| Malecka-Panas | 0.24674822 | 0.19027303 | 0.3032234 |
| Appelros | 0.23801456 | 0.18298946 | 0.29303965 |
| Ibars | 0.24945053 | 0.19224183 | 0.30665922 |
| Malecka-Panas | 0.24752291 | 0.19047752 | 0.30456829 |
| Boreham | 0.24620883 | 0.19011436 | 0.30230328 |
| Halonen | 0.23336445 | 0.18209477 | 0.28463414 |
| Szentkereszty | 0.24756026 | 0.19131908 | 0.30380145 |
| Hochman | 0.24167211 | 0.18607926 | 0.29726496 |
| Kaya | 0.24966651 | 0.19131723 | 0.30801579 |
| Yasuda | 0.23899183 | 0.18383013 | 0.29415351 |
| Pelli | 0.24933235 | 0.19232917 | 0.30633554 |
| Gupta | 0.24542402 | 0.18933243 | 0.30151561 |
| Andersson | 0.24439897 | 0.18836901 | 0.30042893 |
| Uomo | 0.2471206 | 0.19069123 | 0.30354998 |
| Garip | 0.2398096 | 0.18490629 | 0.2947129 |
| Vujasinovic | 0.24846648 | 0.19086799 | 0.30606496 |
| Chandrasekaran | 0.23620547 | 0.18154085 | 0.2908701 |
| Ho | 0.2510674 | 0.19870703 | 0.30342779 |
| Winter Gasparoto | 0.2422065 | 0.18663791 | 0.29777509 |
| Umapathy | 0.2358067 | 0.18188241 | 0.289731 |
| Vipperla | 0.24254648 | 0.1868301 | 0.29826286 |
| Nikkola | 0.24761459 | 0.19097713 | 0.30425206 |
| Tu | 0.24150743 | 0.18616073 | 0.29685411 |
