# Supplementary material for: Incidence of New Onset Diabetes Mellitus Secondary to Acute Pancreatitis: A Systematic Review and Meta-Analysis
Source: Front Physiol. 2019 May 31;10:637. doi: 10.3389/fphys.2019.00637 (PMC6558372; doi:10.3389/fphys.2019.00637)
Supplement: Supplementary file 3 [file Table_3.DOCX]

**Supplementary Table 3. Detailed data on aetiology and severity of AP.**

| Study | Aetiology (%) | | | SAP (%) |
| --- | --- | --- | --- | --- |
|  | Alcoholic | Biliary | Others |  |
| Ohlsen | NR | NR | NR | NR |
| Johansen | 18.2 | 54.5 | 27.3 | NR |
| Olszewski | NR | NR | NR | NR |
| Seligson | 59 | 32.8 | 8.2 | 100 |
| Angelini | 40.7 | 37 | 22.3 | 100 |
| Eriksson | 78 | 6 | 17 | 56 |
| Angelini | 27.1 | 33.1 | 39.8 | 70 |
| Doepel | 75.7 | 8.1 | 16.2 | 100 |
| Malecka-Panas | 100 | 0 | 0 | NR |
| Appelros | 52.6 | 33.4 | 14 | 100 |
| Ibars | 0 | 100 | 0 | 28.6 |
| Malecka-Panas | 43.9 | 35.4 | 20.7 | 34 |
| Boreham | 21.7 | 65.3 | 13 | 30 |
| Halonen | 77.9 | 22.1 | 0 | 100 |
| Szentkereszty | 66.7 | 18.5 | 14.8 | 100 |
| Hochman | 17.1 | 46.3 | 36.6 | 100 |
| Kaya | 7.4 | 55.2 | 37.4 | 63 |
| Yasuda | 51.1 | 22.2 | 26.7 | 100 |
| Pelli | 100 | 0 | 0 | 25 |
| Gupta | 33.3 | 40 | 26.7 | 100 |
| Andersson | 25 | 50 | 25 | 35 |
| Uomo | 0 | 73.7 | 26.3 | 100 |
| Garip | 8.2 | 66 | 25.8 | 35.8 |
| Vujasinovic | 42 | 36 | 22 | 18 |
| Chandrasekaran | 54.3 | 31.4 | 15.2 | 100 |
| Ho | 46.6 | 53.4 | 0 | 6.2 |
| Winter Gasparoto | 25 | 62.5 | 12.5 | 100 |
| Umapathy | 17.9 | 34.3 | 47.8 | 100 |
| Vipperla | NR | 47 | NR | NR |
| Nikkola | NR | NR | NR | 5 |
| Tu | 2.7 | 57.5 | 42.5 | 80.6 |
