# Supplementary material for: Incidence of New Onset Diabetes Mellitus Secondary to Acute Pancreatitis: A Systematic Review and Meta-Analysis
Source: Front Physiol. 2019 May 31;10:637. doi: 10.3389/fphys.2019.00637 (PMC6558372; doi:10.3389/fphys.2019.00637)
Supplement: Supplementary file 4 [file Table_4.DOCX]

**Supplementary Table 4.** Meta-regression for incidence of insulin-treated diabetes after acute pancreatitis

|  | Meta regression coefficient (%) | 95% CI | P |
| --- | --- | --- | --- |
| Male proportion | 0.002 | -0.009 to 0.013 | 0.671 |
| Year of publication | 0.674 | -0.338 to 1.686 | 0.163 |
| Duration of following up | 0.004 | -0.009 to 0.010 | 0.088 |
| Mean age | -0.006 | -0.023 to 0.012 | 0.481 |
