# Supplementary figures and images for: Incidence of New Onset Diabetes Mellitus Secondary to Acute Pancreatitis: A Systematic Review and Meta-Analysis
Source: Front Physiol. 2019 May 31;10:637. doi: 10.3389/fphys.2019.00637 (PMC6558372; doi:10.3389/fphys.2019.00637)

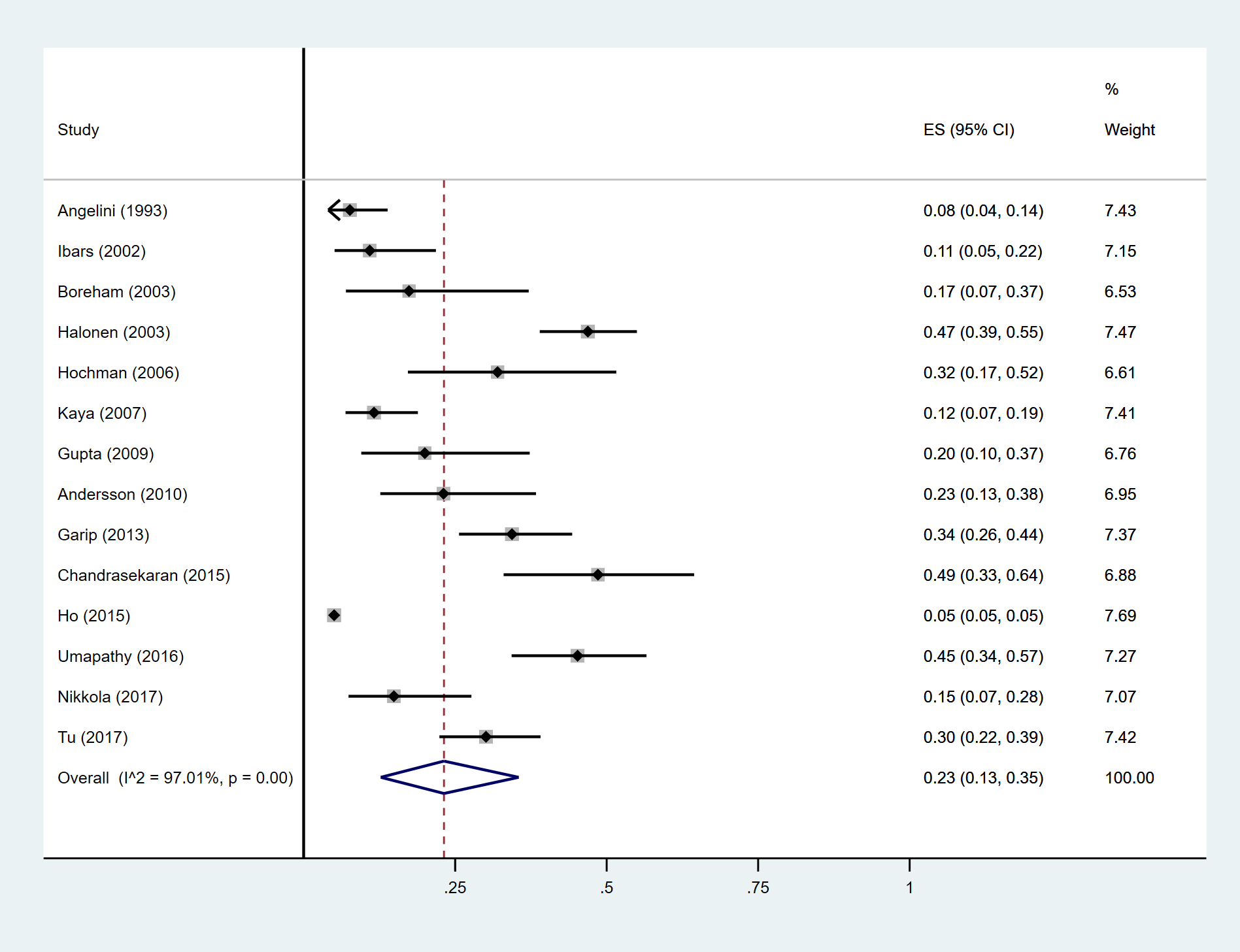

Supplement: Supplementary file 5 [file Image_1.TIF]

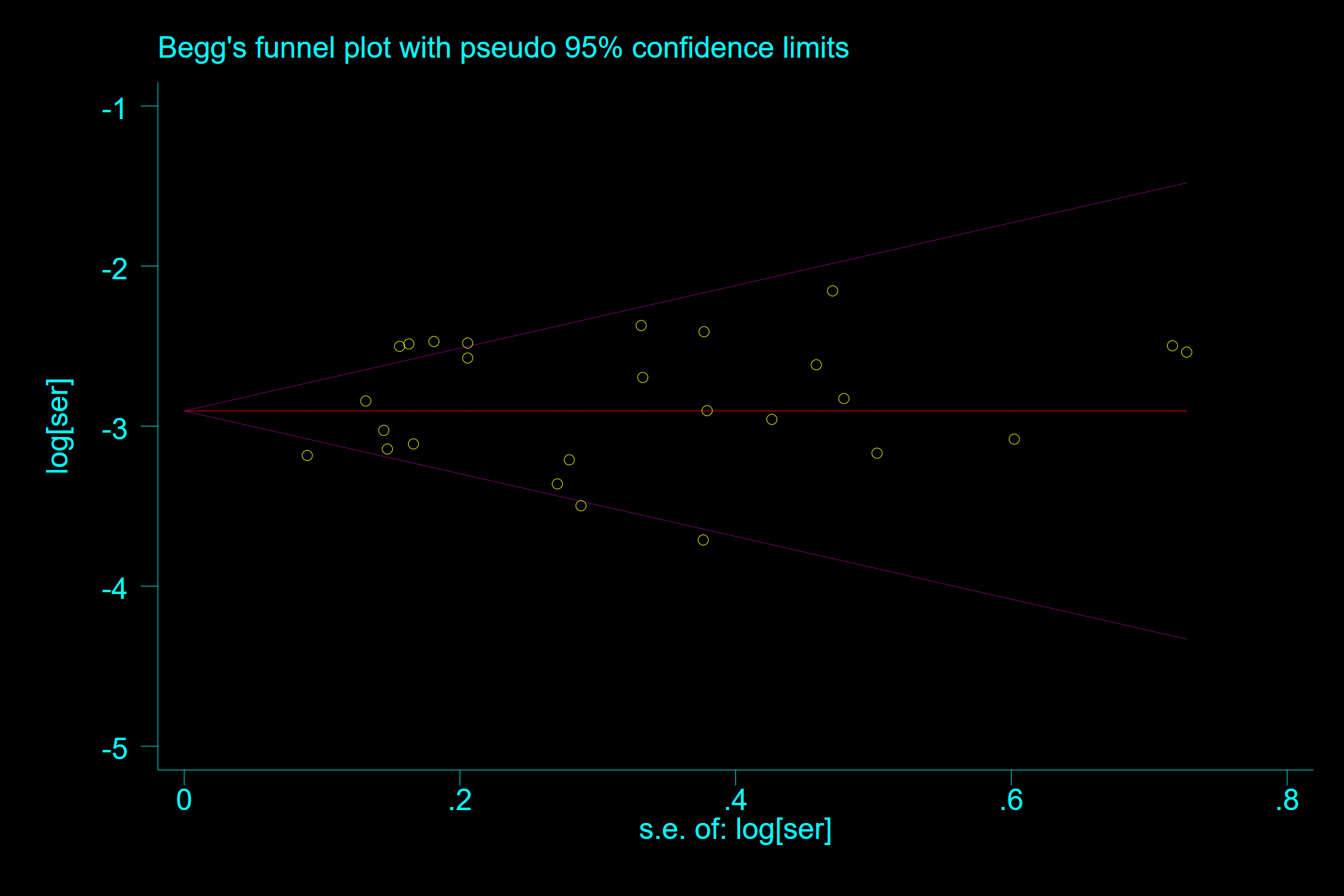

Supplement: Supplementary file 6 [file Image_2.TIF]

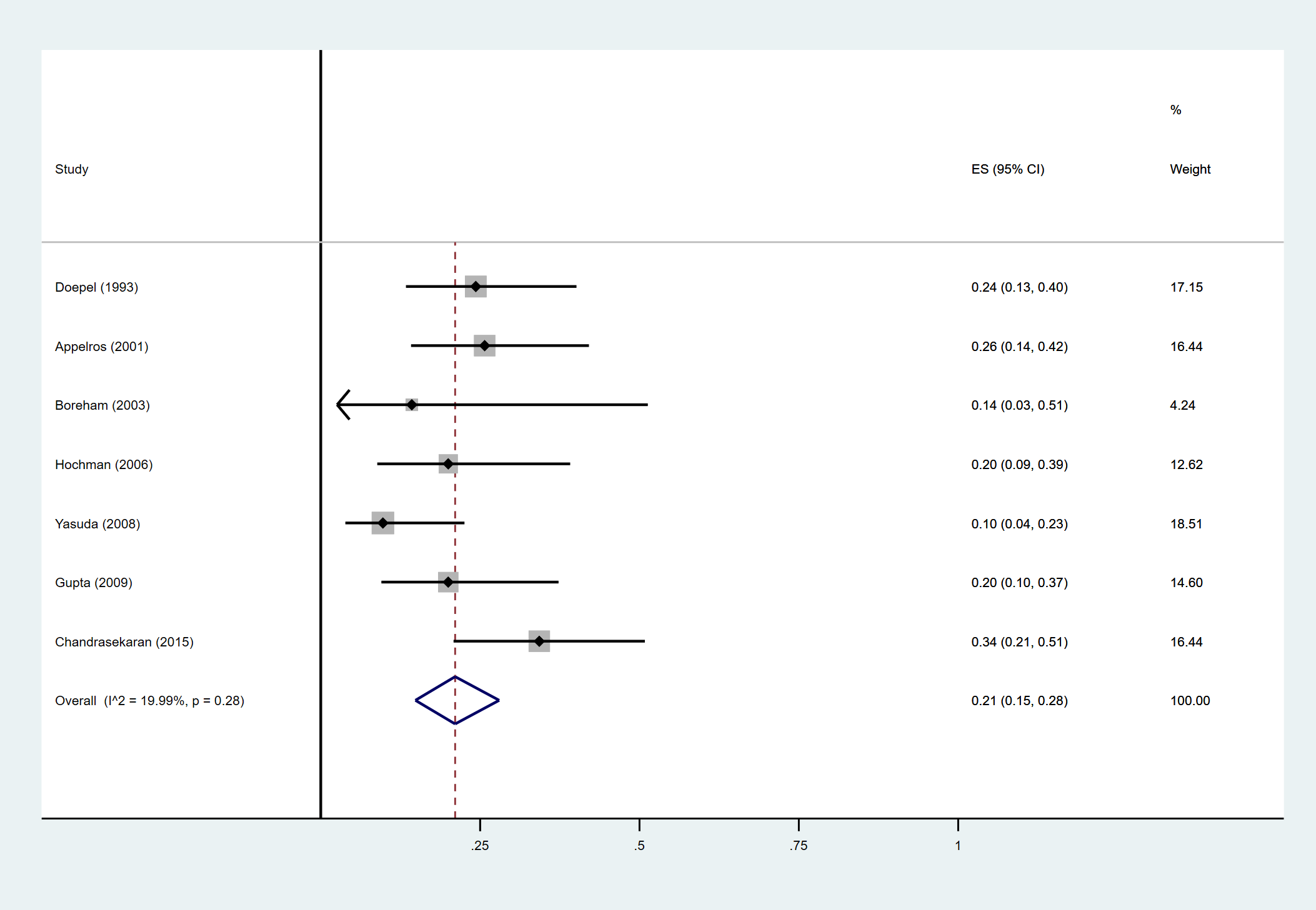

Supplement: Supplementary file 7 [file Image_3.TIF]

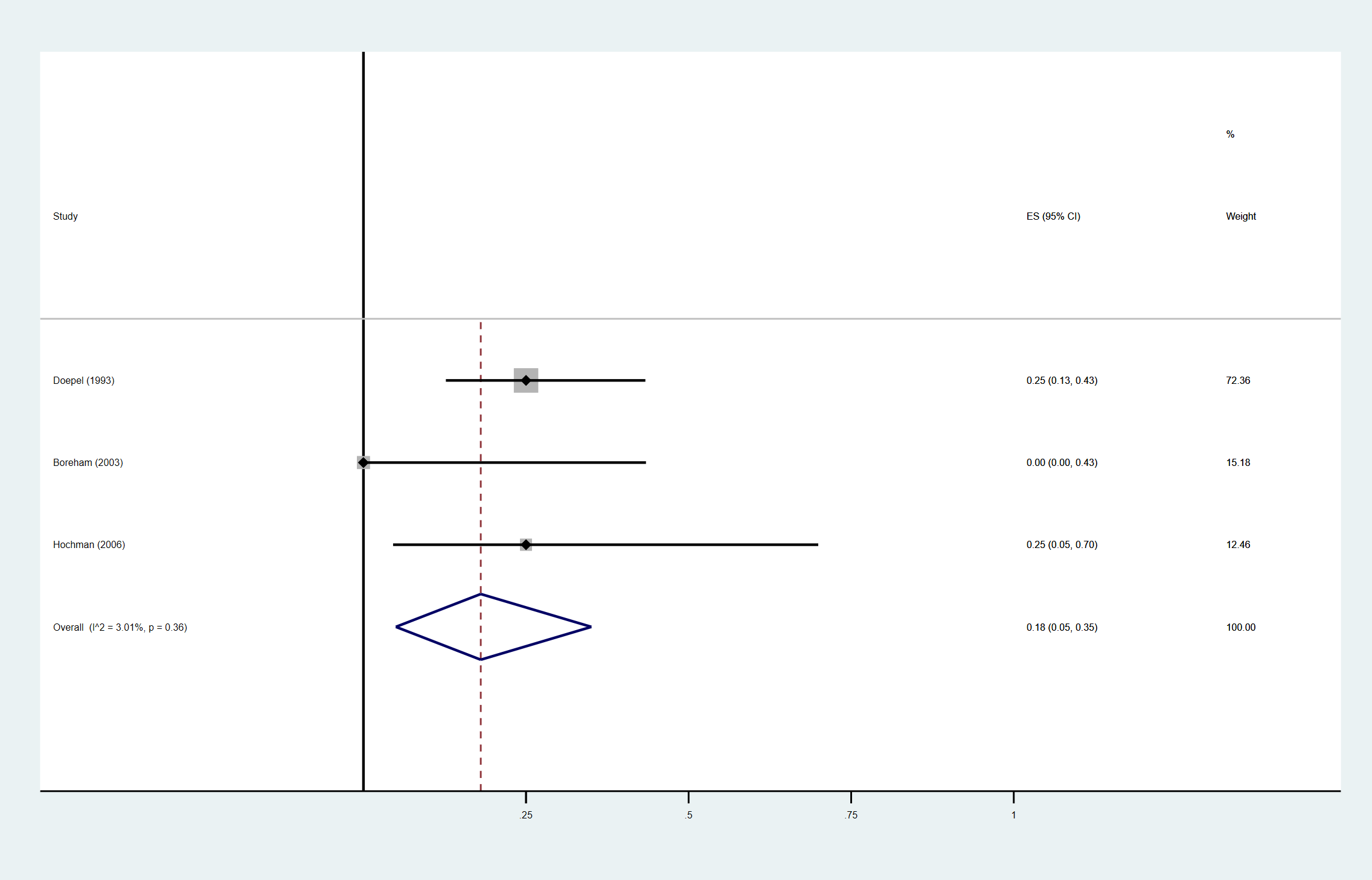

Supplement: Supplementary file 8 [file Image_4.TIF]
